# Developing an implementation research logic model: using a multiple case study design to establish a worked exemplar

**DOI:** 10.1186/s43058-022-00337-8

**Published:** 2022-08-16

**Authors:** Louise Czosnek, Eva M. Zopf, Prue Cormie, Simon Rosenbaum, Justin Richards, Nicole M. Rankin

**Affiliations:** 1 Mary MacKillop Institute for Health Research, Australian Catholic University, Melbourne, Australia; 2 Cabrini Cancer Institute, The Szalmuk Family Department of Medical Oncology, Cabrini Health, Melbourne, Australia; 3 Peter MacCallum Cancer Centre, Melbourne, Australia; 4 Sir Peter MacCallum Department of Oncology, The University of Melbourne, Melbourne, Australia; 5 Discipline of Psychiatry and Mental Health, University of New South Wales, Sydney, Australia; 6 School of Health Sciences, University of New South Wales, Sydney, Australia; 7 Faculty of Health, Victoria University of Wellington, Wellington, New Zealand; 8 Faculty of Medicine and Health, University of Sydney, Sydney, Australia; 9 Faculty of Medicine, Dentistry and Health Sciences, University of Melbourne, Melbourne, Australia

**Keywords:** Logic model, Case study methods, Causal pathways, Causal mechanisms

## Abstract

**Background:**

Implementation science frameworks explore, interpret, and evaluate different components of the implementation process. By using a program logic approach, implementation frameworks with different purposes can be combined to detail complex interactions. The Implementation Research Logic Model (IRLM) facilitates the development of causal pathways and mechanisms that enable implementation. Critical elements of the IRLM vary across different study designs, and its applicability to synthesizing findings across settings is also under-explored. The dual purpose of this study is to develop an IRLM from an implementation research study that used case study methodology and to demonstrate the utility of the IRLM to synthesize findings across case sites.

**Method:**

The method used in the exemplar project and the alignment of the IRLM to case study methodology are described. Cases were purposely selected using replication logic and represent organizations that have embedded exercise in routine care for people with cancer or mental illness. Four data sources were selected: semi-structured interviews with purposely selected staff, organizational document review, observations, and a survey using the Program Sustainability Assessment Tool (PSAT). Framework analysis was used, and an IRLM was produced at each case site. Similar elements within the individual IRLM were identified, extracted, and re-produced to synthesize findings across sites and represent the generalized, cross-case findings.

**Results:**

The IRLM was embedded within multiple stages of the study, including data collection, analysis, and reporting transparency. Between 33-44 determinants and 36-44 implementation strategies were identified at sites that informed individual IRLMs. An example of generalized findings describing “intervention adaptability” demonstrated similarities in determinant detail and mechanisms of implementation strategies across sites. However, different strategies were applied to address similar determinants. Dependent and bi-directional relationships operated along the causal pathway that influenced implementation outcomes.

**Conclusions:**

Case study methods help address implementation research priorities, including developing causal pathways and mechanisms. Embedding the IRLM within the case study approach provided structure and added to the transparency and replicability of the study. Identifying the similar elements across sites helped synthesize findings and give a general explanation of the implementation process. Detailing the methods provides an example for replication that can build generalizable knowledge in implementation research.

**Supplementary Information:**

The online version contains supplementary material available at 10.1186/s43058-022-00337-8.

Contributions to the literature

- Logic models can help understand how and why evidence-based interventions (EBIs) work to produce intended outcomes.
- The implementation research logic model (IRLM) provides a method to understand causal pathways, including determinants, implementation strategies, mechanisms, and implementation outcomes.
- We describe an exemplar project using a multiple case study design that embeds the IRLM at multiple stages. The exemplar explains how the IRLM helped synthesize findings across sites by identifying the common elements within the causal pathway.
- By detailing the exemplar methods, we offer insights into how this approach of using the IRLM is generalizable and can be replicated in other studies.

## Background

The practice of implementation aims to get “someone…, somewhere… to do something differently” [1]. Typically, this involves changing individual behaviors and organizational processes to improve the use of evidence-based interventions (EBIs). To understand this change, implementation science applies different theories, models, and frameworks (hereafter “frameworks”) to describe and evaluate the factors and steps in the implementation process [2–5]. Implementation science provides much-needed theoretical frameworks and a structured approach to process evaluations. One or more frameworks are often used within a program of work to investigate the different stages and elements of implementation [6]. Researchers have acknowledged that the dynamic implementation process could benefit from using logic models [7]. Logic models offer a systematic approach to combining multiple frameworks and to building causal pathways that explain the mechanisms behind individual and organizational change.

Logic models visually represent how an EBI is intended to work [8]. They link the available resources with the activities undertaken, the immediate outputs of this work, and the intermediate outcomes and longer-term impacts [8, 9]. Through this process, causal pathways are identified. For implementation research, the causal pathway provides the interconnection between a chosen EBI, determinants, implementation strategies, and implementation outcomes [10]. Testing causal mechanisms in the research translation pathway will likely dominate the next wave of implementation research [11, 12]. Causal mechanisms (or mechanisms of change) are the “process or event through which an implementation strategy operates to affect desired implementation outcomes” [13]. Identifying mechanisms can improve implementation strategies’ selection, prioritization, and targeting [12, 13]. This provides an efficient and evidence-informed approach to implementation.

Implementation researchers have proposed several methods to develop and examine causal pathways [14, 15] and mechanisms [16, 17]. This includes formalizing the inherent relationship between frameworks via developing the Implementation Research Logic Model (IRLM) [7]. The IRLM is a logic model designed to improve the rigor and reproducibility of implementation research. It specifies the relationship between elements of implementation (determinant, strategies, and outcomes) and the mechanisms of change. To do this, it recommends linking implementation frameworks or relevant taxonomies (e.g., determinant and evaluation frameworks and implementation strategy taxonomy). The IRLM authors suggest the tool has multiple uses, including planning, executing, and reporting on the implementation process and synthesizing implementation findings across different contexts [7]. During its development, the IRLM was tested to confirm its utility in planning, executing, and reporting; however, its utility in synthesizing findings across different contexts is ongoing. Users of the tool are encouraged to consider three principles: (1) comprehensiveness in reporting determinants, implementation strategies, and implementation outcomes; (2) specifying the conceptual relationships via diagrammatic tools such as colors and arrows; and (3) detailing important elements of the study design. Further, the authors also recognize that critical elements of IRLM will vary across different study designs.

This study describes the development of an IRLM from a multiple case study design. Case study methodology can answer “how and why” questions about implementation. They enable researchers to develop a rich, in-depth understanding of a contemporary phenomenon within its natural context [18–21]. These methods can create coherence in the dynamic context in which EBIs exist [22, 23]. Case studies are common in implementation research [24–30], with multiple case study designs suitable for undertaking comparisons across contexts [31, 32]. However, they are infrequently applied to establish mechanisms [11] or combine implementation elements to synthesize findings across contexts (as possible through the IRLM). Hollick and colleagues [33] undertook a comparative case study, guided by a determinant framework, to explore how context influences successful implementation. The authors contrasted determinants across sites where implementation was successful versus sites where implementation failed. The study did not extend to identifying implementation strategies or mechanisms. By contrast, van Zelm et al. [31] undertook a theory-driven evaluation of successful implementation across ten hospitals. They used joint displays to present mechanisms of change aligned with evaluation outcomes; however, they did not identify the implementation strategies within the causal pathway. Our study seeks to build on these works and explore the utility of the IRLM in synthesizing findings across sites. The dual objectives of this paper were to:

- Describe how case study methods can be applied to develop an IRLM
- Demonstrate the utility of the IRLM in synthesizing implementation findings across case sites.

## Method

In this section, we describe the methods used in the exemplar case study and the alignment of the IRLM to this approach. The exemplar study explored the implementation of exercise EBIs in the context of the Australian healthcare system. The exemplar study aimed to investigate the integration of exercise EBIs within routine mental illness or cancer care. The evidence base detailing the therapeutic benefits of exercise for non-communicable diseases such as cancer and mental illness are extensively documented [34–36] but inconsistently implemented as part of routine care [37–44].

Additional file 1 provides the Standards for Reporting Qualitative Research (SRQR).

### Case study approach

We adopted an approach to case studies based on the methods described by Yin [18]. This approach is said to have post-positivist philosophical leanings, which are typically associated with the quantitative paradigm [19, 45, 46]. This is evidenced by the structured, deductive approach to the methods that are described with a constant lens on objectivity, validity, and generalization [46]. Yin’s approach to case studies aligns with the IRLM for several reasons. The IRLM is designed to use established implementation frameworks. The two frameworks and one taxonomy applied in our exemplar were the Consolidated Framework for Implementation Research (CFIR) [47], Expert Recommendations for Implementing Change (ERIC) [48], and Proctor et al.’s implementation outcomes framework [49]. These frameworks guided multiple aspects of our study (see Table 1). Commencing an implementation study with a preconceived plan based upon established frameworks is deductive [22]. Second, the IRLM has its foundation in logic modeling to develop cause and effect relationships [8]. Yin advocates using logic models to analyze case study findings [18]. They argue that developing logic models encourages researchers to iterate and consider plausible counterfactual explanations before upholding the causal pathway. Further, Yin notes that case studies are particularly valuable for explaining the transitions and context within the cause-and-effect relationship [18]. In our exemplar, the transition was the mechanism between the implementation strategy and implementation outcome. Finally, the proposed function of IRLM to synthesize findings across sites aligns with the exemplar study that used a multiple case approach. Multiple case studies aim to develop generalizable knowledge [18, 50].

**Table 1:** Theoretical application within the study and operational definitions/measures for implementation outcomes

Framework	Application to research study		
**Consolidated Framework for Implementation Research (CFIR)**	The CFIR is applied in this study to identify and describe the determinants that influenced implementation at each site. The semi-structured interview guide was developed with reference to the *CFIR interview building tool*. During data analysis, each CFIR construct was established as a code and as examples were identified, they were indexed to the relevant determinant.		
**Expert Recommendations for Implementing Change (ERIC)**	The ERIC taxonomy is applied in the study to develop consistent descriptions of the actions undertaken at each case site used to facilitate implementation. The *ERIC—discrete implementation strategy compilation with ancillary material (Additional file 6)—*provided a reference tool that aided data collection and interpretation of data sources. During data analysis, each ERIC strategy was applied as a code. As examples were identified, they were indexed to the relevant ERIC strategy.		
**Implementation outcomes framework**	The Implementation Outcomes Framework defines 8 implementation outcomes, of which this study focused on 4: acceptability, fidelity, penetration, and sustainability. Each implementation outcome was operationalized and a measure defined which guided data collection (see below). During data analysis each implementation outcome was established as a code and as examples were identified, they were indexed to the relevant implementation outcome.		
	**Operational definition for implementation outcome**	**Measure**	
*Acceptability*	How is exercise EBI perceived in the organization?	Direct question	
*Fidelity*	Comparison of exercise EBI protocol with what is delivered as measured by dose/amount and quality of program delivery	*Dose/amount* *Quality*	Sessions/duration per week Total program delivered versus intended amount (dose delivered) Attendance rates (dose received) Evidence of training to support delivery
*Penetration*	Measured at the service level (reach) and at the organizational system level (policies/procedures that evidence the EBI)	*Reach* *System level penetration*	Number of people who use the service versus total population Evidence of job descriptions, budget, and strategic plans that reference the exercise EBI
*Sustainability*	Evidence of continued health benefit, program components and evolution overtime	*Health benefits* *Program components* *Evolution*	Process exits to measure health benefits in patients Survey (PSAT) Survey (PSAT)

*EBI* evidence-based intervention, *PSAT* Program Sustainability Assessment Tool, *CFIR* Consolidated Framework for Implementation Research, *ERIC* Expert Recommendations for Implementing Change

### Case study selection and boundaries

A unique feature of Yin’s approach to multiple case studies is using replication logic to select cases [18]. Cases are chosen to demonstrate similarities (literal replication) or differences for anticipated reasons (theoretical replication) [18]. In the exemplar study, the cases were purposely selected using literal replication and displayed several common characteristics. First, all cases had delivered exercise EBIs within normal operations for at least 12 months. Second, each case site delivered exercise EBIs as part of routine care for a non-communicable disease (cancer or mental illness diagnosis). Finally, each site delivered the exercise EBI within the existing governance structures of the Australian healthcare system. That is, the organizations used established funding and service delivery models of the Australian healthcare system.

Using replication logic, we posited that sites would exhibit some similarities in the implementation process across contexts (literal replication). However, based on existing implementation literature [32, 51–53], we expected sites to adapt the EBIs through the implementation process. The determinant analysis should explain these adaptions, which is informed by the CFIR (theoretical replication). Finally, in case study methods, clearly defining the boundaries of each case and the units of analysis, such as individual, the organization or intervention, helps focus the research. We considered each healthcare organization as a separate case. Within that, organizational-level analysis [18, 54] and operationalizing the implementation outcomes focused inquiry (Table 1).

### Data collection

**Fig. 1:**
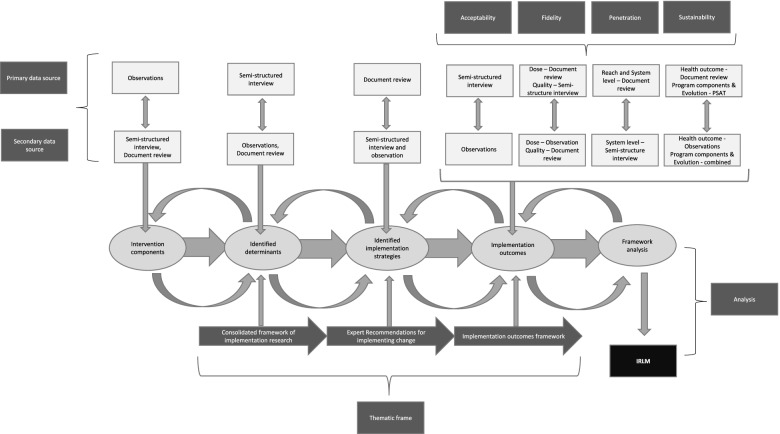
Conceptual frame for the study

During the study conceptualization for the exemplar, we mapped the data sources to the different elements of the IRLM (Fig. 1). Four primary data sources informed data collection: (1) semi-structured interviews with staff; (2) document review (such as meeting minutes, strategic plans, and consultant reports); (3) naturalistic observations; and (4) a validated survey (Program Sustainability Assessment Tool (PSAT)). A case study database was developed using Microsoft Excel to manage and organize data collection [18, 54].

### Semi-structured interviews

An interview guide was developed, informed by the CFIR interview guide tool [55]. Questions were selected across the five domains of the CFIR, which aligned with the delineation of determinant domains in the IRLM. Purposeful selection was used to identify staff for the interviews [56]. Adequate sample size in qualitative studies, particularly regarding the number of interviews, is often determined when data saturation is reached [57, 58]. Unfortunately, there is little consensus on the definition of saturation [59], how to interpret when it has occurred [57], or whether it is possible to pre-determine in qualitative studies [60]. The number of participants in this study was determined based on the staff’s differential experience with the exercise EBI and their role in the organization. This approach sought to obtain a rounded view of how the EBI operated at each site [23, 61]. Focusing on staff experiences also aligned with the organizational lens that bounded the study. Typical roles identified for the semi-structured interviews included the health professional delivering the EBI, the program manager responsible for the EBI, an organizational executive, referral sources, and other health professionals (e.g., nurses, allied health). Between five and ten interviews were conducted at each site. Interview times ranged from 16 to 72 min, most lasting around 40 min per participant.

### Document review

A checklist informed by case study literature was developed outlining the typical documents the research team was seeking [18]. The types of documents sought to review included job descriptions, strategic plans/planning documents, operating procedures and organizational policies, communications (e.g., website, media releases, email, meeting minutes), annual reports, administrative databases/files, evaluation reports, third party consultant reports, and routinely collected numerical data that measured implementation outcomes [27]. As each document was identified, it was numbered, dated, and recorded in the case study database with a short description of the content related to the research aims and the corresponding IRLM construct. Between 24 and 33 documents were accessed at each site. A total of 116 documents were reviewed across the case sites.

### Naturalistic observations

The onsite observations occurred over 1 week, wherein typical organizational operations were viewed. The research team interacted with staff, asked questions, and sought clarification of what was being observed; however, they did not disrupt the usual work routines. Observations allowed us to understand how the exercise EBI operated and contrast that with documented processes and procedures. They also provided the opportunity to observe non-verbal cues and interactions between staff. While onsite, case notes were recorded directly into the case study database [62, 63]. Between 15 and 40 h were spent on observations per site. A total of 95 h was spent across sites on direct observations.

### Program sustainability assessment tool (survey)

The PSAT is a planning and evaluation tool that assesses the sustainability of an intervention across eight domains [64–66]: (1) environmental support, (2) funding stability, (3) partnerships, (4) organizational capacity, (5) program evaluation, (6) program adaption, (7) communication, and (8) strategic planning [64, 65]. The PSAT was administered to a subset of at least three participants per site who completed the semi-structured interview. The results were then pooled to provide an organization-wide view of EBI sustainability. Three participants per case site are consistent with previous studies that have used the tool [67, 68] and recommendations for appropriate use [65, 69].

We included a validated measure of sustainability, recognizing calls to improve understanding of this aspect of implementation [70–72]. Noting the limited number of measurement tools for evaluating sustainability [73], the PSAT’s characteristics displayed the best alignment with the study aims. To determine “best alignment,” we deferred to a study by Lennox and colleagues that helps researchers select suitable measurement tools based on the conceptualization of sustainability in the study [71]. The PSAT provides a multi-level view of sustainability. It is a measurement tool that can be triangulated with other implementation frameworks, such as the CFIR [74], to interrogate better and understand the later stages of implementation. Further, the tool provides a contemporary account of an EBIs capacity for sustainability [75]. This is consistent with case study methods, which explore complex, contemporary, real-life phenomena.

The voluminous data collection that is possible through case studies, and is often viewed as a challenge of the method [19], was advantageous to developing the IRLM in the exemplar and identifying the causal pathways. First, it aided three types of triangulation through the study (method, theory, and data source triangulation) [76]. Method triangulation involved collecting evidence via four methods: interview, observations, document review, and survey. Theoretical triangulation involved applying two frameworks and one taxonomy to understand and interpret the findings. Data source triangulation involved selecting participants with different roles within the organization to gain multiple perspectives about the phenomena being studied. Second, data collection facilitated depth and nuance in detailing determinants and implementation strategies. For the determinant analysis, this illuminated the subtleties within context and improved confidence and accuracy for prioritizing determinants. As case studies are essentially “naturalistic” studies, they provide insight into strategies that are implementable in pragmatic settings. Finally, the design’s flexibility enabled the integration of a survey and routinely collected numerical data as evaluation measures for implementation outcomes. This allowed us to contrast “numbers” against participants’ subjective experience of implementation [77].

### Data analysis

Descriptive statistics were calculated for the PSAT and combined with the three other data sources wherein framework analysis [78, 79] was used to analyze the data. Framework analysis includes five main phases: familiarization, identifying a thematic framework, indexing, charting, and mapping and interpretation [78]. *Familiarization* occurred concurrently with data collection, and the *thematic frame* was aligned to the two frameworks and one taxonomy we applied to the IRLM. To *index and chart* the data, the raw data was uploaded into NVivo 12 [80]. Codes were established to guide indexing that aligned with the thematic frame. That is, determinants within the CFIR [47], implementation strategies listed in ERIC [48], and the implementation outcomes [49] of acceptability, fidelity, penetration, and sustainability were used as codes in NVivo 12. This process produced a framework matrix that summarized the information housed under each code at each case site.

The final step of framework analysis involves *mapping and interpreting* the data. We used the IRLM to map and interpret the data in the exemplar. First, we identified the core elements of the implemented exercise EBI. Next, we applied the CFIR valance and strength coding to prioritize the contextual determinants. Then, we identified the implementation strategies used to address the contextual determinants. Finally, we provided a rationale (a causal mechanism) for how these strategies worked to address barriers and contribute to specific implementation outcomes. The systematic approach advocated by the IRLM provided a transparent representation of the causal pathway underpinning the implementation of the exercise EBIs. This process was followed at each case site to produce an IRLM for each organization. To compare, contrast, and synthesize findings across sites, we identified the similarities and differences in the individual IRLMs and then developed an IRLM that explained a generalized process for implementation. Through the development of the causal pathway and mechanisms, we deferred to existing literature seeking to establish these relationships [81–83]. Aligned with case study methods, this facilitated an iterative process of constant comparison and challenging the proposed causal relationships. Smith and colleagues advise that the IRLM “might be viewed as a somewhat simplified format,” and users are encouraged to “iterate on the design of the IRLM to increase its utility” [7]. Thus, we re-designed the IRLM within a traditional logic model structure to help make sense of the data collected through the case studies. Figure 1 depicts the conceptual frame for the study and provides a graphical representation of how the IRLM pathway was produced.

## Results

The results are presented with reference to the three principles of the IRLM: *comprehensiveness, indicating the key conceptual relationship* and *specifying critical study design*. The case study method allowed for comprehensiveness through the data collection and analysis described above. The mean number of data sources informing the analysis and development of the causal pathway at each case site was 63.75 (interviews (*M* = 7), observational hours (*M*=23.75), PSAT (*M*=4), and document review (*M* = 29). This resulted in more than 30 determinants and a similar number of implementation strategies identified at each site (determinant range per site = 33–44; implementation strategy range per site = 36–44). Developing a framework matrix meant that each determinant (prioritized and other), implementation strategy, and implementation outcome were captured. The matrix provided a direct link to the data sources that informed the content within each construct. An example from each construct was collated alongside the summary to evidence the findings.

**Fig. 2:**
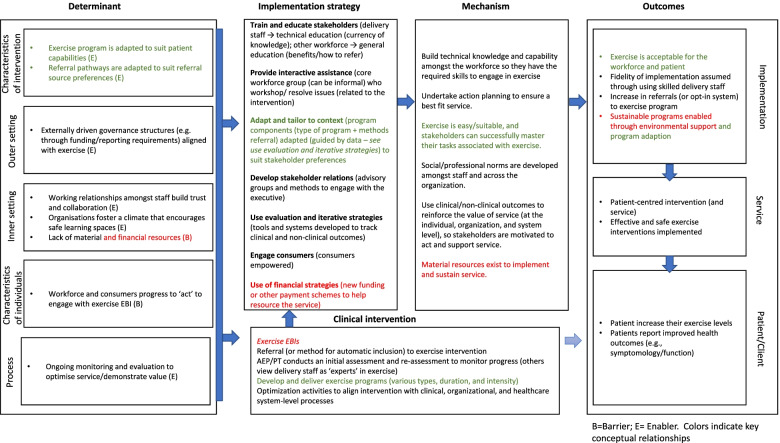
Example of intervention adaptability (E) contrasted with available resources (B) within a synthesised IRLM across case sites

The *key conceptual relationship* was articulated in a traditional linear process by aligning determinant → implementation strategy → mechanism → implementation outcome, as per the IRLM. To synthesize findings across sites, we compared and contrasted the results within each of the individual IRLM and extracted similar elements to develop a generalized IRLM that represents cross-case findings. By redeveloping the IRLM within a traditional logic model structure, we added visual representations of the bi-directional and dependent relationships, illuminating the dynamism within the implementation process. To illustrate, intervention adaptability was a prioritized determinant and enabler across sites. Healthcare providers recognized that adapting and tailoring exercise EBIs increased “fit” with consumer needs. This also extended to adapting how healthcare providers referred consumers to exercise so that it was easy in the context of their other work priorities. Successful adaption was contingent upon a qualified workforce with the required skills and competencies to enact change. Different implementation strategies were used to make adaptions across sites, such as promoting adaptability and using data experts. However, despite the different strategies, successful adaptation created positive bi-directional relationships. That is, healthcare providers’ confidence and trust in the EBI grew as consumer engagement increased and clinical improvements were observed. This triggered greater engagement with the EBI (e.g., acceptability → penetration → sustainability), albeit the degree of engagement differed across sites. Figure 2 illustrates this relationship within the IRLM and provides a contrasting relationship by highlighting how a prioritized barrier across sites (available resources) was addressed.

The final principle is to *specify critical study design*, wherein we have described how case study methodology was used to develop the IRLM exemplar. Our intention was to produce an explanatory causal pathway for the implementation process. The implementation outcomes of acceptability and fidelity were measured at the level of the provider, and penetration and sustainability were measured at the organizational level [49]. Service level and clinical level outcomes were not identified for a priori measurement throughout the study. We did identify evidence of clinical outcomes that supported our overall findings via the document review. Historical evaluations on the service indicated patients increased their exercise level or demonstrated a change in symptomology/function. The implementation strategies specified in the study were those chosen by the organizations. We did not attempt to augment routine practice or change implementation outcomes by introducing new strategies. The barriers across sites were represented with a (B) symbol and enablers with an (E) symbol in the IRLM. In the individual IRLM, consistent determinants and strategies were highlighted (via bolding) to support extraction. Finally, within the generalized IRLM, the implementation strategies are grouped according to the ERIC taxonomy category. This accounts for the different strategies applied to achieve similar outcomes across case studies.

## Discussion

This study provides a comprehensive overview that uses case study methodology to develop an IRLM in an implementation research project. Using an exemplar that examines implementation in different healthcare settings, we illustrate how the IRLM (that documents the causal pathways and mechanisms) was developed and enabled the synthesis of findings across sites.

Case study methodologies are fraught with inconsistencies in terminology and approach. We adopted the method described by Yin. Its guiding paradigm, which is rooted in objectivity, means it can be viewed as less flexible than other approaches [46, 84]. We found the approach offered sufficient flexibility within the frame of a defined process. We argue that the defined process adds to the rigor and reproducibility of the study, which is consistent with the principles of implementation science. That is, accessing multiple sources of evidence, applying replication logic to select cases, maintaining a case study database, and developing logic models to establish causal pathways, demonstrates the reliability and validity of the study. The method was flexible enough to embed the IRLM within multiple phases of the study design, including conceptualization, philosophical alignment, and analysis. Paparini and colleagues [85] are developing guidance that recognizes the challenges and unmet value of case study methods for implementation research. This work, supported by the UK Medical Research Council, aims to enhance the conceptualization, application, analysis, and reporting of case studies. This should encourage and support researchers to use case study methods in implementation research with increased confidence.

The IRLM produced a relatively linear depiction of the relationship between context, strategies, and outcomes in our exemplar. However, as noted by the authors of the IRLM, the implementation process is rarely linear. If the tool is applied too rigidly, it may inadvertently depict an overly simplistic view of a complex process. To address this, we redeveloped the IRLM within a traditional logic model structure, adding visual representations of the dependent and bidirectional relationships evident within the general IRLM pathway [86]. Further, developing a general IRLM of cross-case findings that synthesized results involved a more inductive approach to identifying and extracting similar elements. It required the research team to consider broader patterns in the data before offering a prospective account of the implementation process. This was in contrast to the earlier analysis phases that directly mapped determinants and strategies to the CFIR and ERIC taxonomy. We argue that extracting similar elements is analogous to approaches that have variously been described as portable elements [87], common elements [88], or generalization by mechanism [89]. While defined and approached slightly differently, these approaches aim to identify elements frequently shared across effective EBIs and thus can form the basis of future EBIs to increase their utility, efficiency, and effectiveness [88]. We identified similarities related to determinant detail and mechanism of different implementation strategies across sites. This finding supports the view that many implementation strategies could be suitable, and selecting the “right mix” is challenging [16]. Identifying common mechanisms, such as increased motivation, skill acquisition, or optimizing workflow, enabled elucidation of the important functions of strategies. This can help inform the selection of appropriate strategies in future implementation efforts.

Finally, by developing individual IRLMs and then re-producing a general IRLM, we synthesized findings across sites and offered generalized findings. The ability to generalize from case studies is debated [89, 90], with some considering the concept a fallacy [91]. That is, the purpose of qualitative research is to develop a richness through data that is situated within a unique context. Trying to extrapolate from findings is at odds with exploring unique context. We suggest the method described herein and the application of IRLM could be best applied to a form of generalization called ‘transferability’ [91, 92]. This suggests that findings from one study can be transferred to another setting or population group. In this approach, the new site takes the information supplied and determines those aspects that would fit with their unique environment. We argue that elucidating the implementation process across multiple sites improves the confidence with which certain “elements” could be applied to future implementation efforts. For example, our approach may also be helpful for multi-site implementation studies that use methods other than case studies. Developing a general IRLM through study conceptualization could identify consistencies in baseline implementation status across sites. Multi-site implementation projects may seek to introduce and empirically test implementation strategies, such as via a cluster randomized controlled trial [93]. Within this study design, baseline comparison between control and intervention sites might extend to a comparison of organizational type, location and size, and individual characteristics, but not the chosen implementation strategies [94]. Applying the approach described within our study could enhance our understanding of how to support effective implementation.

### Limitations

After the research team conceived this study, the authors of the PSAT validated another tool for use in clinical settings (Clinical Sustainability Assessment Tool (CSAT)) [95]. This tool appears to align better with our study design due to its explicit focus on maintaining structured clinical care practices. The use of multiple data sources and consistency in some elements across the PSAT and CSAT should minimize the limitations in using the PSAT survey tool. At most case sites, limited staff were involved in developing and implementing exercise EBI. Participants who self-selected for interviews may be more invested in assuring positive outcomes for the exercise EBI. Inviting participants from various roles was intended to reduce selection bias. Finally, we recognize recent correspondence suggesting the IRLM misses a critical step in the causal pathway. That is the mechanism between determinant and selection of an appropriate implementation strategy [96]. Similarly, Lewis and colleagues note that additional elements, including pre-conditions, moderators, and mediators (distal and proximal), exist within the causal pathway [13]. Through the iterative process of developing the IRLM, decisions were made about the determinant → implementation strategy relationship; however, this is not captured in the IRLM. Secondary analysis of the case study data would allow elucidation of these relationships, as this information can be extracted through the case study database. This was outside the scope of the exemplar study.

## Conclusion

Developing an IRLM via case study methods proved useful in identifying causal pathways and mechanisms. The IRLM can complement and enhance the study design by providing a consistent and structured approach. In detailing our approach, we offer an example of how multiple case study designs that embed the IRLM can aid the synthesis of findings across sites. It also provides a method that can be replicated in future studies. Such transparency adds to the quality, reliability, and validity of implementation research.

## Supplementary Information

**Additional file 1.** Standards for Reporting Qualitative Research (SRQR).

## Data Availability

The data that support the findings of this study are available on request from the corresponding author [LC]. The data are not publicly available due to them containing information that could compromise research participant privacy.
